# Combined Nasal, Oropharyngeal Povidone Iodine Plus Glycyrrhizic Acid Sprays, Accelerate Clinical and Laboratory Recovery and Reduces Household Transmission of SARS-CoV-2: A Randomized Placebo-Controlled Clinical Trial

**DOI:** 10.3389/fmed.2022.863917

**Published:** 2022-04-19

**Authors:** Hazem E. Elsersy, Magdy A. H. Zahran, Abd-Elazeem Elbakry, Mohamed Abd-Elwahab, Mohamed Milegy Ahmed, Mohamed Salah Elgandy, Eman H. M. Mohammed, Nourhan M. Elewa

**Affiliations:** ^1^Department of Anesthesia and Intensive Care, Faculty of Medicine, Menoufia University, Shebin El-Kom, Egypt; ^2^Organic Chemistry Department, Faculty of Science, Menoufia University, Shebin El-Kom, Egypt; ^3^Department of Chest Diseases, Faculty of Medicine, Tanta University, Tanta, Egypt; ^4^Internal Medicine Department, Liver Institute, Menoufia University, Shebin El-Kom, Egypt; ^5^Ear, Nose, and Throat Department, Faculty of Medicine, Zagazig University, Zagazig, Egypt

**Keywords:** acid glycyrrhizic, iodine povidone, antiviral, SARS-CoV-2, nasal spray, oropharyngeal spray

## Abstract

The COVID-19 pandemic is still posing challenging health and economic problems. Effective broad-spectrum antiviral therapy is urgently needed for the control of early SARS-CoV-2 infection to limit its spread and mutations. In this randomized placebo-controlled clinical study, we tested the effects of intranasal and oropharyngeal delivery of a compound of povidone-iodine 0.5% and glycyrrhizic acid 2.5 mg/ml on the laboratory (PCR) and clinical recovery from SARS-CoV-2 patients and their household contacts. 353 patients suspected of having COVID-19 infection were screened by chest CT and nasopharyngeal swab tests (PCR). 200 patients were randomly allocated to two equal groups: treatment and placebo groups. Treatment accelerated the recovery of PCR on days 4, 7, and 10, as evidenced by PCR-positive patients (70, vs. 99%, 20 vs. 65%, 1 vs. 10%) in both the treated and placebo groups, respectively. Treatment enhanced the early recovery of symptoms [day 7.6 ± 2 (CI 7:8.3) vs. 8.9 ± 2 (CI 8.3:9.6)]. Treatment promoted early recovery of anosmia and ageusia [5.6 ± 1 (CI, 4.8:6.4) vs. 11 ± 3 days, (CI, 10.8:12)] in both the treated and control groups (*P* < 0.0001). There was a notable reduction in transmission of the virus among the household close contacts in the treatment group (4%) vs. 76% in the placebo group. Combined PVI-GA nasal and oropharyngeal spray accelerates both laboratory and clinical recovery of SARS-CoV-2 infected patients in the early phases of the disease and reduces the household spread of the virus; thus, it may play an important role in controlling coronavirus outbreaks.

**Clinical Trial Registration:**

https://pactr.samrc.ac.za, PACTR202101875903773.

## Introduction

The COVID-19 pandemic is considered a huge health and economic problem affecting the entire world population. Current treatment protocols use systemic antiviral agents, immunostimulants, or vaccines (1); however, to date, no satisfactory treatment regimen has been developed, and the number of people dying because of COVID-19 is still rising. From the pathophysiologic perspective, the course of COVID-19 disease can be stratified into two distinctive phases. Phase 1 corresponds to the incubation period and the early symptomatic phase wherein the virus still resides in the nasopharynx and oropharynx (2), and phase 2 corresponds to the phase in which the virus invades the lower respiratory tract. The appearance of novel highly infective strains such as Delta and Omicron represents a real challenge to the permanence of vaccine protection (3, 4). This issue urges the discovery of broad-spectrum antivirals that can help with vaccines to terminate the pandemic.

It is intuitively attractive to consider the use of multiple broad-spectrum antivirals that can be applied as topical sprays to abort early infected cases with the SARS-CoV-2 virus. The combination of glycyrrhizic acid (GA) and povidone-iodine (PVI) for topical spraying seems to be a good candidate for several reasons. First, both GA and PVI have been shown to attenuate and kill the novel coronavirus *in vitro* by several different mechanisms (5, 6).

Second, the anti-inflammatory and demulcent effects of GA might ameliorate the irritant effects of iodine on both nasal and oral mucosa (7). Third, the combination of two or more antiviral compounds would allow the use of a lower dose of each, thus reducing their adverse reactions.

Povidone-iodine 0.5% has been shown to rapidly inactivate the novel coronavirus in 15 s *in vitro* (5). This concentration can be used safely as a nasal and nasopharyngeal spray and is safe to apply to mucosal goblet cells (5). Glycyrrhizic acid has been proven to effectively neutralize the SARS-CoV-2 virus *in vitro* at subtoxic concentrations (8). Povidone-iodine mouth wash and nasal spray have been shown to reduce the viral load in patients with COVID-19 infection (9).

The purpose of the current investigation is to use a novel multimodal antiviral combination to attack the virus early in its incubation period and early clinical phases to reduce the viral load and viral multiplication, thus reducing the spread to the lower respiratory tree, preventing pneumonia and ICU admission and speeding the recovery of the patients.

## Methods

These studies were conducted by professors from Menoufia, Tanta and Zagazig universities in Egypt and were approved by the General Fever and Liver Institute local Ethics Committee in Shebin Alkom, Menoufia, Egypt. The study has been registered according to WHO and ICMJ standards in the PACTR registry under the number PACTR202101875903773. All studies including compound preparation and pilots were conducted between March 2020 through July 2021. The trial protocol of this study is included in the attached Supplementary Materials.

### Studies Design

Pilot studies 1, 2, 3, and 4 were designed to test tolerability, efficacy on SARS-CoV-2 PCR, the occurrence of side effects, and protection of household contacts from getting COVID-19 infection [see Supplementary Material]

#### The Randomized-Blinded Controlled Study

Two hundred patients of both genders aged 18 to 80 years old who tested positive for COVID-19 with early symptoms of coronavirus and had free CT chest, in addition to their household contacts, were recruited in the study. Informed consent was obtained from each patient. Permuted block randomization was carried out after the PCR results using a computer-generated research randomizer program into two equal parallel groups: the treatment group and the placebo group. Household contacts aged 6 to 80 were treated in accordance with their patient allocation. For randomization purposes, each patient together with his household contacts were considered to represent one case study, where either the treatment or placebo medications were given to the patient and his contacts. The exclusion criteria were patients with CT evidence of lung invasion, patients with dropped O_2_ saturation below 90 on room air, patients with thyroid disorders, uncontrolled hypertension or diabetes mellitus, and ventilated patients or those with multiorgan failure.

Patients in the treatment group received standard hospital treatment in the form of vitamin C, paracetamol, and zinc, plus both nasal and oropharyngeal spray bottles. The spray active ingredients included a compound of glycyrrhizic acid in the form of ammonium glycyrrhizate 2.5 mg/ml plus PVI 0.5% for oropharyngeal and dipotassium glycyrrhizinate 2.5 mg/ml Plus PVI 0.5% for nasal spray (PVI- AL nil Co., under authority of Mundi-pharma). GA and its salts were purchased from Sigma Aldrich pharmaceutical grade USA standards, USA. The Menoufia Faculty of Science team developed a simple effective and non-toxic method of extraction of glycyrrhizic acid and its salts. [Supplementary Material].

The placebo group received standard hospital treatment plus placebo oropharyngeal and nasal sprays. The medications were prepared by the pharmacist, physician doctor and chemist in collaboration between members from the faculty of science, faculty of medicine, Menoufia University. The compound was packed in opaque medical-grade plastic bottles. An excipient flavoring agent and suitable preservative were added. The bottles were labeled and encoded by the pharmacist who blinded investigators, outcome assessors and the patients. Patient allocation was performed by central randomization through phone calls to the pharmacist, and codes were kept confidential by the pharmacist until the last day of follow-up for the last patient.

#### Patient Consent and Instructions

Patients with a positive PCR test for COVID-19 with or without symptoms but with negative chest CT findings were seen and examined at the outpatient infection control clinics in the fever and liver hospital. The number of household contacts was recorded, and consent from contacts or their guardians if below 21 years of age was obtained. Instructions for use consisted of a tutorial on how to use and the frequency of the use of sprays by the patients. Patients were advised to concomitantly use oropharyngeal and nasal sprays 6 times per day. They were instructed to abstain from food, drink, and smoke for 20 min, particularly after oropharyngeal spray. The oropharyngeal spray bottle contains an atomizer that ends with a long arm applicator to insert inside the mouth cavity and can be directed up, down, right, or left to cover the entire pharyngeal area.

### Outcome Assessment

All patient data, such as age, sex, body weight, O2 saturation, blood pressure, pulse, and associated disease, were recorded and compared between the two groups.

#### The Primary Outcomes

The primary outcome for the patients was the SARS-CoV-2 polymerase chain reaction (PCR) recovery rate. PCR was tested on days 4, 7, and 10 following the start of the treatment using a nasopharyngeal swab (Genesig® Real-Time PCR assay, PrimerdesignTM Ltd).

The primary outcome for the contacts was the number of household contacts who developed COVID-19 clinical symptoms within 15 days from confirmed PCR-positive family members and the start of treatment. COVID-19 clinical symptoms included fever, cough, body aches, running nose, headache, vertigo, diarrhea, loss of taste and smell. Contacts who developed clinical symptoms suspecting COVID-19 were tested with PCR to confirm or refute infection. A household was defined as two or more persons living together in the same indoor living space.

#### The Secondary Outcomes

##### The Day Symptoms Disappear

Patients were followed daily through a home visit in the morning and two phone calls per day for the improvement of their symptoms, and the day on which the last symptom disappeared was recorded and compared between the groups.

##### The Day of the Return of Taste and/or Smell

This particular symptom was followed as a separate item, and the day of full return of function was confirmed by tasting with a known flavor sweet and sour object and smelling of characteristic perfume. The function was considered regained if the patient reported that it returned to baseline. For statistical analysis purposes, the data for taste and smell patients were analyzed by excluding patients who did not develop anosmia and ageusia. Therefore, a subgroup of patients who developed anosmia and ageusia were compared.

##### Development of Complications

such as desaturation, pneumonia, hospitalization, ICU admission or death was followed and recorded for 1 month from initiation of treatment.

### Statistical Analysis

#### Sample Size Calculation

Based on our pilot study with PCR for COVID-19 as the primary determinant of sample size, given an effect size of 50% as a difference between the means, Student's *t*-test and given a power of 95% and alpha error of 0.05 revealed that 88 patients were required for each group with a total sample size of 176. We increased the sample size to 200, 100 per group, to avoid a drop in the sample size due to possible exclusions. Power calculation was performed using the G-Power 3.1.9.4 program protocol for power analysis.

#### Statistical Testing

Descriptive statistics were used to analyze the patient demographic data, such as age, sex, and baseline oxygen saturation. Two-tailed Student's *t*-test was used to compare the day symptoms disappeared and the day of recovery of taste and smell. Because the PCR variable data were either positive or negative (discrete data), the total PCR data are expressed as percentages and numbers and were analyzed by chi-square testing. Confidence intervals between the means or medians were estimated, and a *P*-value <0.05 was considered significant.

Statistical analysis was performed using the XL STAT program for Windows (Addinosoft, exe, 2016).

## Results

**Figure 1:**
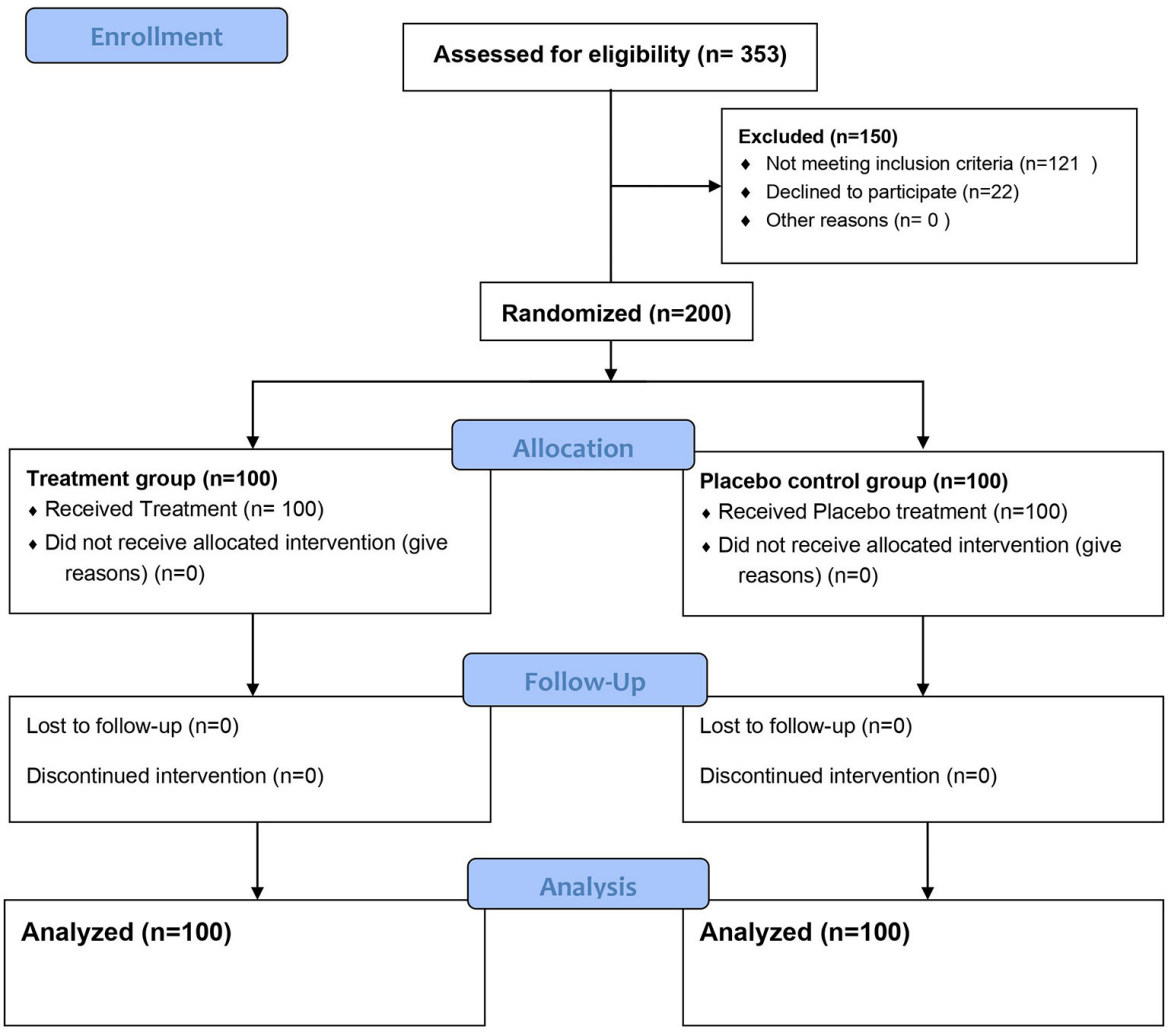
Consort study diagram.

**Figure 2:**
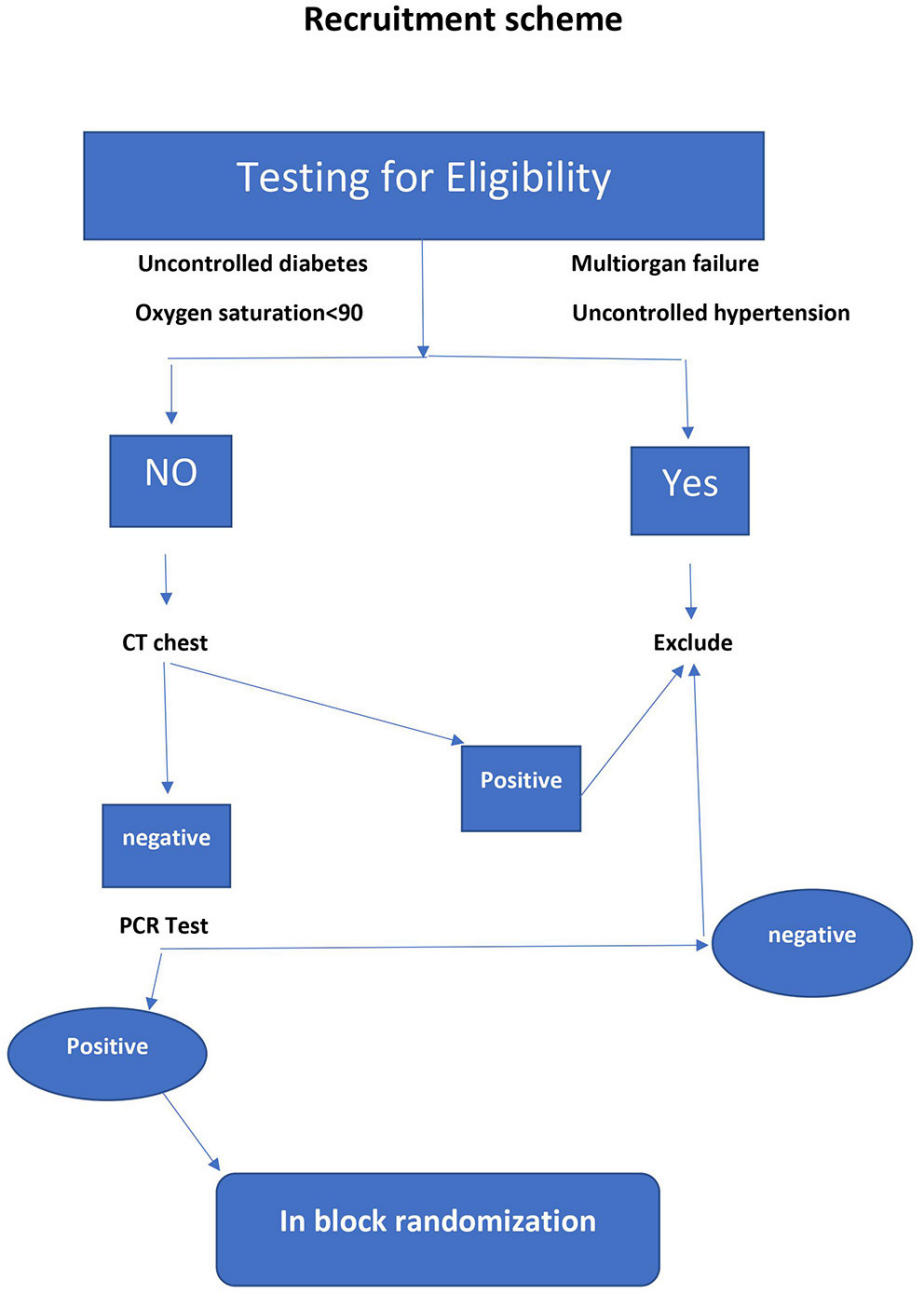
Recruitment scheme.

Figures 1, 2 represent a schematic diagram of recruitment, randomization, and the allocation process of the patients. Three hundred fifty-three patients were tested for eligibility, 153 were excluded; 121 did not meet the inclusion criteria, and 32 patients declined to participate. Thus, 200 patients were randomized, received interventions, and were followed from March through July 2021. There was no difference between the groups in patient characteristics (Table 1).

**Table 1:** Patient characteristics and clinical symptoms and signs.

**Characteristic**	**Treatment group**	**Placebo group**
Age (years) [median (min–max)]	35.5 (20–68)	36.5 (18–70)
Gender [Males/Females]	(M/F) 38/62	(M/F) 46/54
BMI (kg/m^2^)	31.5 ± 4	31 ± 3
Pulse (beat/min) [M ± SD]	75 ± 4	76 ± 8
SBP (mmHg) [M ± SD]	121 ± 8	120 ± 14
DBP (mmHg) [M ± SD]	76 ± 6	77 ± 6
SPO2 (%) [Med. IQR]	98 (97–98)	97.5 (97–98)
Temperature (Celsius) [Med IQR]	38 (38–39)	38 (38–38.2)
Cough no (%)	72 (72%)	60 (60%)
Body ache no (%)	93 (93%)	91 (91%)
Anosmia no (%)	59 (59%)	64 (64%)
Ageusia no (%)	56 (56%)	53 (53%)
Headache no (%)	12 (12%)	17 (17%)
Vomiting no (%)	7 (7%)	5 (5%)
Diarrhea no (%)	17 (17%)	10 (10%)
Vertigo no (%)	4 (4%)	2 (2%)
Fever no (%)	90 (90%)	86 (86%)
No of household contacts	194	277
Side effects	Throat irritation 2 (2%)	No throat irritation

*Patient characteristics. Values are expressed as numbers and percentages; medians and interquartile ranges or means ± standard deviations*.

*M/F, Male to female; BMI, Body mass index; (kg/m^2^), Kilogram/ square meter; SPO2, Oxygen saturation; SBP, Systolic blood pressure; DBP, Diastolic blood pressure; NO, Number; (Min-Max), Minimum- Maximum; [M ± SD], Mean ± standard deviation; [Med. IQR], Median; interquartile range; (%), Percentage*.

**Figure 3:**
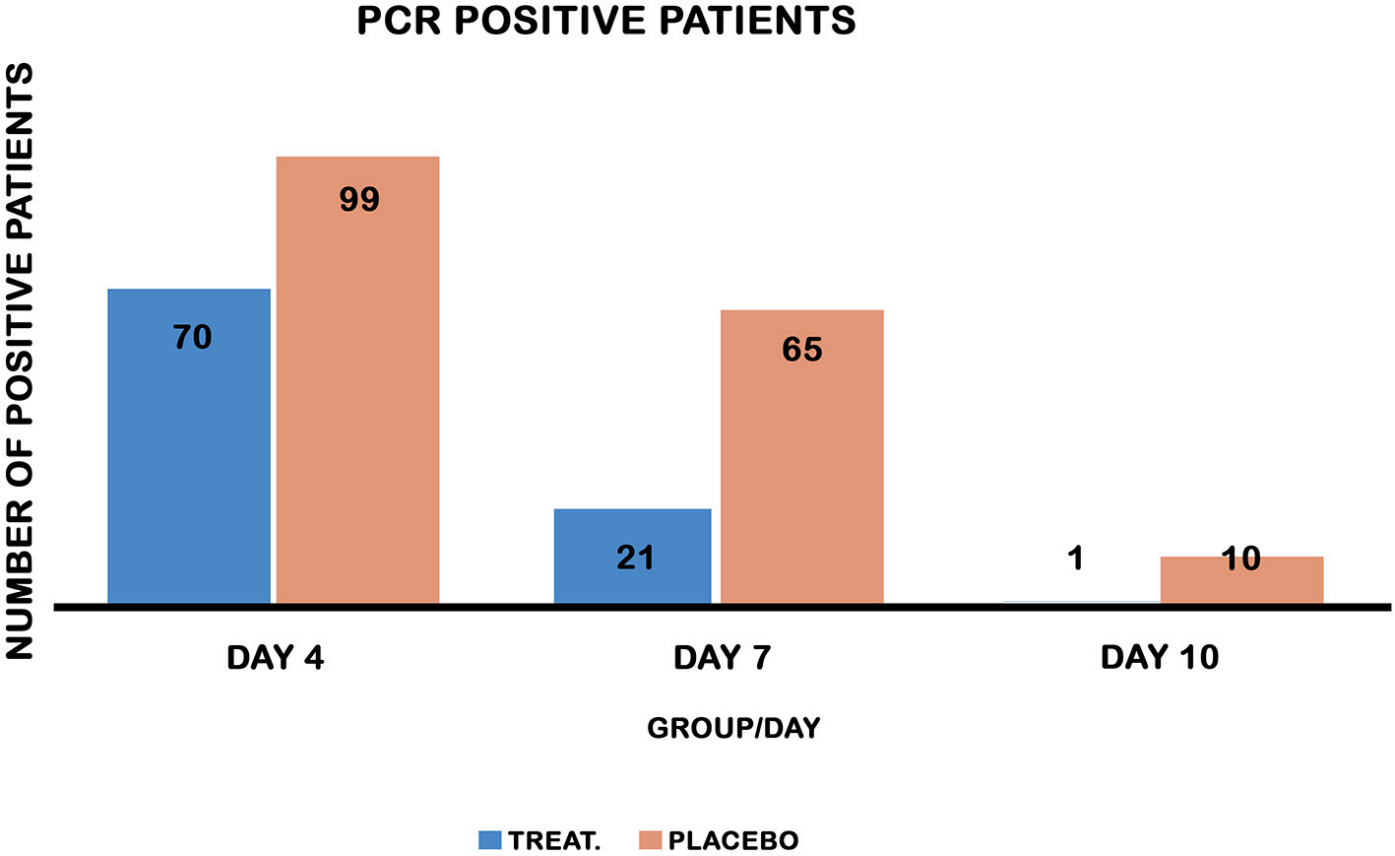
PCR-positive patients on days 4, 7, and 10 in both groups. Chi-square test comparing the recovery of PCR in both treatment and placebo control groups. Treatment reduced PCR-positive patients on days 4, 7, and 10 post-treatment, *P* values [ <0.0001, 0.001, and 0.005] respectively.

Chi-square testing showed that the treatment group displayed a reduction in the number of PCR-positive patients on days 4, 7, and 10 compared to the placebo control group (Table 2, Figure 3). On day 4, a striking number of patients were PCR negative relative to the placebo control (30 vs. 1%) and 95% lower to upper boundary interval [9:22] and (9–23) in both the treated and placebo groups, respectively. On day 7, the recovery of PCR was 79 vs. 35%, 95% lower to upper boundary interval [46–74] and [46–70] in both the treatment and placebo groups, respectively. PCR measured on day 10 posttreatment revealed a recovery of 99% of the treated vs. 90% of the placebo (Table 2).

**Table 2:** PCR for patients and positive contacts and percentage of complications of both treatment and placebo groups.

**Variable**	**Treatment group**	**Placebo group**	***P*-value**
Day 4 PCR positive/negative (number)	70/30 [73:99]^*^/[8:22]^**^	99/1 [70:97]^*^/[9-22]^**^	<0.0001
Day 7 PCR positive/negative (number)	21/79 [32:54]^*^/[44:70]^**^	65/35 [32:53]^*^/[44-71]^**^	0.001
Day 10 PCR positive/negative (number)	1/99[1–9]^*^/[81:109]^**^	10/90 [1:9]^*^/[81:107]^**^	0.005
Covid-19 symptoms Positive contacts	12/194 (6%)	173/227 (76%)	<0.0001
Covid-19 PCR positive contacts	8/194 (4%)	157/227 (69%)	<0.0001
Percentage of spared contacts	(94: 96%)	(24:31%)	
% of complications	0%	7%	

*Shows the study outcome variables. Data are expressed as numbers and percentages.*

* *Displays the 95% upper and lower boundary for PCR-positive patients, while*

** *reflects the 95% upper and lower boundaries for PCR-negative patients. PCR, polymerase chain reaction; %, percentage. PCR-positive patients were compared using the chi-square test*.

Subgroup analysis for anosmia and ageusia and for other symptoms indicated that the day of complete recovery from COVID-19 symptoms was earlier than that of the control group, where the mean recovery day was 7.6 ± 2, CI around the mean [7:8.3] vs. 8.9 ± 2, CI around the mean [8.3:9.6] days in both the treatment and placebo control groups, respectively, and CI for the difference between the means = [−2.2: −0.4] (Table 3).

**Table 3:** Recovery of symptoms from COVID-19.

**variable**	**Treatment group**	**Placebo group**	**CI (FDBM)**	***P*-value**
DSD (mean ± SD) [CI around the mean]	7.6 ± 2 [7: 8.3]	8.9 ± 2[8.3-9.6]	[−2.2: −0.4]	0.008
Recovery of Smell (day post-treatment)	5.6 ± 1.3[ 4.8:6.4]	11 ± 3.4 [10.8:12.1]	[−6.9: −4.6]	<0.0001
Recovery of taste (day post-treatment)	5.7 ± 1[4.8:6.5]	11 ± 4 [10.5:12.3]	[−6.8: −4.4]	<0.0001

*Displays the day of recovery of symptoms of COVID-19 in both the treated and placebo groups. Data are expressed as the mean ± standard deviation, showing the confidence interval around the means and the confidence intervals for the difference between the means. CI (FDBM), Confidence intervals for the difference between the means; DSD, Day symptom disappear; (mean ± SD), Mean ± standard deviation*.

**Figure 4:**
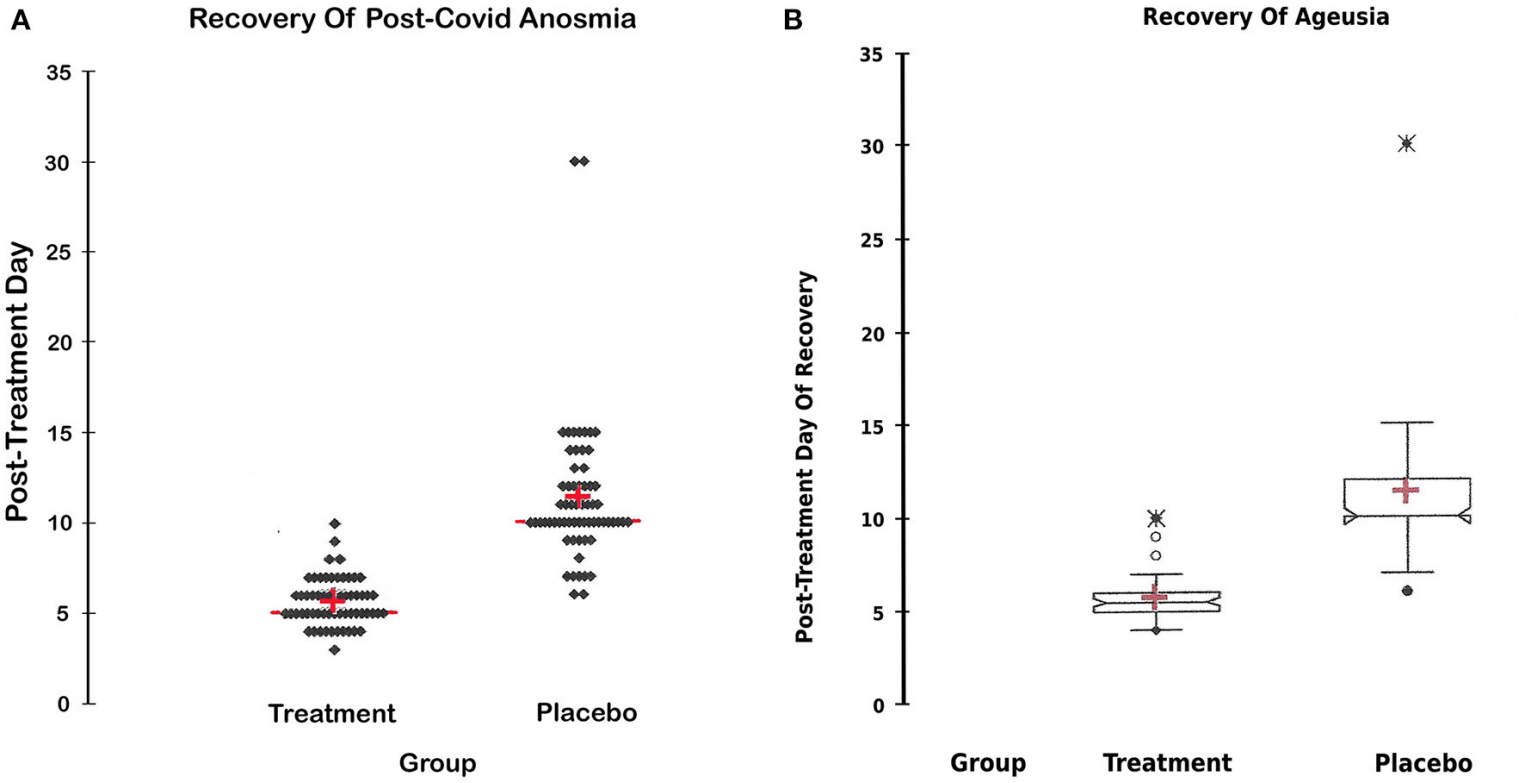
**(A)** Recovery of anosmia. **(A)** Day of the return of smell in both groups; the day of complete recovery of lost smell after commencement of treatment in both groups. A statistical figure (scattergram) where the cross represents the mean, the transverse red line represents the median, the upper and lower dots represent the minimum and maximum, and each dot represents a patient recovery timing. The treatment restored lost smell significantly earlier than the placebo group CI around the mean [4.6:6.9], *P*-value <0.0001. **(B)** Recovery of Ageusia. **(B)** Day of the return of taste in both groups; the day of complete recovery of lost taste sensation after commencement of treatment in both groups. A statistical figure where the cross represents the mean, the upper and lower dots represent the minimum and maximum, and the notches represent the confidence interval around the medium. The treatment restored lost smell significantly earlier than the placebo group, CI around the mean [4.4:6.9], *P* < 0.0001.

The overall incidence of anosmia was 61.5%, while the incidence of ageusia was 54.5% in both groups. Figures 4A,B display the day of complete recovery of taste and smell. Treatment with the sprays restored taste and smell significantly earlier than the placebo control (5.6 ± 1 vs. 11 ± 3 days), CI around the means [4.6:6.9] for anosmia and [4.4:6.9] for ageusia, *P* < 0.0001 (Table 3).

To reveal the difference in response to the effect of treatment on gender and age a subgroup analysis with respect to these two variables revealed, an effect of treatment on the primary outcome variable in both males and females compared to the placebo subgroup on post-treatment days 4 and 7 PCR. Similarly, there was an effect of treatment on both younger and elder age groups (Table 4). With respect to day 10 post-treatment PCR, there was a small effect of gender on the outcome where there was no difference between the treatment and placebo in females, *P* = 0.064 while there was a difference between treated and placebo male groups *p* = 0.036. In addition, the effect of treatment was obvious on post-treatment day 10 in the elder age group, *P* = 0.023 while, this effect was not noticed in the younger age group *P* = 0.087 (Table 4).

**Table 4:** Effect of treatment on age and gender groups.

**Variables**	**Treat. +ve**	**Placeb. +ve**	**Treat. –ve**	**Placeb. –ve**	***P*-value**
Males PCR4	25	45	13	1	<0.0001
Females PCR4	17	54	45	0	<0.0001
Males PCR7	5	33	33	13	<0.0001
Females PCR7	22	32	46	16	0.001
Males PCR 10	0	5	38	41	0.036
Females PCR 10	1	5	61	49	0.064
Young PCR 4	36	48	14	1	0.001
Old PCR4	34	51	16	0	<0.0001
Young PCR 7	12	28	38	21	0.002
Old PCR 7	9	37	41	14	<0.0001
Young PCR10	1	5	49	44	0.087
Old PCR10	0	5	50	46	0.023

*Displays the effect of the treatment on males and females, young and old age groups. Subgroup analysis was carried out by Chi-square testing. Young PCR, age group from 18–35 years; the old PCR, age group from 36 to 70. Treat, treatment; Placeb, Placebo; +ve, Positive; –ve, negative; PCR 4, 7, and 10, Polymerase chain reaction on day 4, 7, and 10 post-treatment*.

In the present study, the overall incidence of secondary infection transmission among household contacts was found to be 41% in both groups. From a clinical symptom perspective, treatment with PVI-GA sprays resulted in a reduction in the transmission rate from 76 to 6% among the studied patient groups, while according to PCR testing, treatment reduced SARS-CoV-2 transmission from 69 to 4%; therefore, in the treatment arm, 94:96% of patients were protected from becoming infected (Table 2).

The placebo control group displayed a 7% rate of complications (Table 2), where five patients developed hypoxemia and dyspnea, dropped in SPO2, and were hospitalized, and two patients did not restore their taste and smell during the study period (long COVID-19). In the treatment group, there were no reported complications. Other than 2 patients (2%) who developed mild throat irritation, there were no noticed or reported side effects due to treatment with sprays throughout the study period. No ICU admissions or deaths occurred during the study period in either group.

## Discussion

The main findings of the present study are that treatment with PVI in combination with GA nasal and oropharyngeal spray resulted in early laboratory and clinical recovery of patients suffering COVID-19 infection. In addition, the use of sprays has substantially reduced the transmission of COVID-19 among household direct contacts of known cases.

PVI has been shown to rapidly inactivate SARS-CoV-2 *in vitro* in <15 s (5) and to reduce the viral load in the nasopharynx for patients treated with nasal and oral sprays (9). Similarly, glycyrrhizic acid has effectively neutralized SARS-CoV-2 *in vitro* by inhibiting the virus's main protease (8).

We are not aware of studies that used topical glycyrrhizic acid for nasal or oropharyngeal sprays or studies that used a combination of both drugs in the treatment and prevention of the spread of SARS-CoV-2 or other respiratory viruses.

It can be predicted that a drug that has a notable effect *in vitro* will have such an effect *in vivo* when it is used as a topical application more likely than if it is applied systematically. This is because systemically applied drugs undergo significant alterations when they are exposed to gastric juice, hepatic first bypass metabolism, plasma protein binding, and distribution issues (10). The environment created by tissue culture could mimic the nasal and oral mucosa to a great extent. For instance, oral glycyrrhizic acid is converted to glycyrrhetic acid (more toxic) by intestinal commensal bacteria, where glycyrrhizic acid itself becomes indetectable in the plasma (11, 12).

Nasal goblet and ciliated cells have the highest expression of angiotensin-converting enzyme 2 (ACE2), which is the main receptor for SARS-CoV-2 (13). Recent research shows a strong interaction of SARS-CoV-2 with human ACE2 (14, 15). Letko et al. reported that the SARS-CoV-2 receptor-binding domain could enter cells expressing human host cell ACE2, not any of the other receptors, confirming that human ACE2 is the receptor for the recently emerging SARS-CoV-2 (16). Glycyrrhizic acid has been shown to reduce SARS-CoV-2 virus replication *in vitro* by multiple mechanisms (17, 18). Glycyrrhizic acid has recently been shown to have the potential to bind to ACE2 (19). Glycyrrhizic acid reduces virus transmission by reducing the expression of type 2 transmembrane serine protease (TMPRSS2), which is required for virus uptake (6, 18). Glycyrrhizic acid may reduce the severity of COVID-19 infection at the two stages of the COVID-19-induced disease process, block the number of entry points (ACE2 receptors) and provide an ACE2-independent anti-inflammatory mechanism (18).

One of the most important and surprising findings of the present study is the rapid recovery of taste and smell sensations in the treatment group. Recently, an association between the novel coronavirus and the development of anosmia and ageusia has been documented in many countries worldwide (19). The exact mechanism underlying anosmia and ageusia in COVID-19 infection is still unclear (20); however, a proposal of viral invasion of the olfactory bulb and olfactory receptor cells has been postulated (21). Brann et al. reported that the sustentacular cells that support sensory neurons in the nose are the target for virus infection, entering through ACE2 receptors on the cell surface (21). Cazzolla et al. reported evidence of inflammatory processes in the olfactory bulb of patients infected with COVID-19. Therefore, in light of the current available body of evidence, it is tempting to speculate that glycyrrhizic acid, which possesses anti-inflammatory properties and works upon ACE2 receptors, played a pivotal role in speeding the recovery of olfactory and gustatory dysfunction caused by SARS-CoV-2 infection in the current study (22).

Glycyrrhizic acid has broad-spectrum antiviral activity and has inhibitory effects on herpes simplex, hepatitis C, Coxiakie, influenza virus with its subtypes, and SARS-CoV-2 virus (23).

It is unlikely that nasal PVI alone can cure early cases of coronavirus. While PVI possesses strong antiviral, antibacterial, and antifungal effects, it lacks anti-inflammatory action (24). This defect is compensated for by the addition of GA, which possesses broad-spectrum antiviral and strong anti-inflammatory action, making it a perfect combination to fight SARS-CoV-2 and its effects. The dose was kept far below 2 mg/kg, which is the dose of zero side effects of glycyrrhizic acid (25). The safety of glycyrrhizic acid and its salts has been documented and approved by the FDA (26).

COVID-19 patients have been shown to transmit the virus during their incubation period. Wang et al. reported that the highest risk of household transmission occurs prior to symptom onset (27). It has been shown that the viral load is at its peak 2 days before symptom onset and on the first day of symptoms, and up to 44% of transmission occurs during the presymptomatic period in settings with substantial household clustering. (28, 29). Therefore, with the absence of frequent routine testing, it is hard to predict who must be isolated to avoid the spread of the virus. Asymptomatic household contacts may be in their incubation periods and can pose a risk to other family members or outdoor communities. In the present study, the secondary transmission rate among the placebo-treated patients was 76% (69% as confirmed by PCR), which is relatively high. In our society, intimate family relations and the reluctance to use protective equipment, such as wearing masks at home, may have contributed to this high incidence.

In the present study, treatment with sprays cut the vicious circle and substantially reduced SARS-CoV-2 transmission among direct-household contacts. This effect can be interpreted in two ways: first, reducing the viral load in the patient to reduce transmission to the contacts. Second, decontamination of the nasopharyngeal passages of the contacts and reduction of their viral loads give a chance to their immune system to overcome the invader. This finding has a relevant clinical impact in limiting the viral spread and giving a hand to the vaccine in patients who are allergic or intolerant to the vaccine or those in whom vaccination is contraindicated.

This study showed that both males and females, young and old responded equally to the treatment with small differences on day 10 PCR that may be due to the earlier recovery of female gender and younger age sub-groups in the placebo group. It is possible that the marked reduction of the compared number of the patients by dividing them into four subgroups has contributed to this observation. Further studies are needed to address this issue.

In the present study, the complication rate was 7% among the placebo control group, and none had ICU admission or died. This issue may be due to the patient population selected, where we excluded high-risk patients. Nevertheless, treatment with sprays has completely prevented the occurrence of complications.

Our study has several strengths. It is a randomized prospective, placebo-controlled study with an adequately powered sample size. In addition, it uncovers a novel route of administration to glycyrrhizic acid and provides a novel combination of topically applied antiviral compounds that may possess a broad-spectrum antiviral effect that is not limited to the COVID-19 virus alone. Third, the meticulous choice of the treated patient population who are positive in PCR but negative in chest CT findings provided clear indications for the use of this drug combination.

The study has some limitations. First, we did not quantify the viral load in the nasal passages; however, a recent clinical investigation showed a reduction in coronavirus load following nasal PVI (9). Second, household secondary infection may not be limited to indoor sources, as with other studies, community outdoor infection cannot be rolled out. We did not perform variant-specific testing, as the PCR used identifies only SARS-CoV-2 infection but does not differentiate alpha from delta strains. Finally, not all contacts were subjected to PCR testing, and only those contacts who developed symptoms were tested for PCR. The reasons for this are the lack of standard methods that determine when to perform a PCR test for asymptomatic contact to prove or refute the infection; therefore, only those contacts who developed clinically suspicious symptoms underwent PCR testing. Further studies are planned among higher-risk patients to test whether the addition of a systemic oral glycyrrhizic acid in combination with sprays can go further in a more advanced stage of the disease.

## Conclusions

The use of a combination of povidone-iodine 0.5% and glycyrrhizic acid 2.5 mg/ml as nasal, oral, and pharyngeal sprays might accelerate SARS-CoV-2 PCR and symptom recovery, restore lost taste and smell early, and may offer good protection for household contacts of early mild cases of SARS-CoV-2 patients.

## Data Availability Statement

The original contributions presented in the study are included in the article/Supplementary Material, further inquiries can be directed to the corresponding author.

## Ethics Statement

The studies involving human participants were reviewed and approved by Fever and Liver Hospital Local Ethics Committee. Written informed consent to participate in this study was provided by the patients/participants or their legal guardian/next of kin.

## Author Contributions

HE: Idea, intellectual property, protocol design, experimental design, data analysis, manuscript writing, pilot 1, and pilot 2 design and conduction. MZ: Data analysis, manuscript writing, extraction, and purification of Glycyrrhizic acid and its salts. A-EE: Experimental design, data analysis, manuscript writing, randomization, and allocation. MA-E: Experimental design, data analysis, Manuscript writing, design, and conduction of pilot 3. MA: Data analysis, Manuscript writing design, and conduction of pilot 4. ME: Data analysis, manuscript writing, and case report conduction. EM and NE: Data analysis, Manuscript writing, Glycyrrhizic acid, and its salts extraction. All authors contributed to the article and approved the submitted version.

## Funding

The present study was funded by donations from Elsersy Scientific Med. company.

## Conflict of Interest

This project has been filed as a patent application for HE, international PCT PCT/EG/2021000030, national application number 2020/1599. The remaining authors declare that the research was conducted in the absence of any commercial or financial relationships that could be construed as a potential conflict of interest.

## Publisher's Note

All claims expressed in this article are solely those of the authors and do not necessarily represent those of their affiliated organizations, or those of the publisher, the editors and the reviewers. Any product that may be evaluated in this article, or claim that may be made by its manufacturer, is not guaranteed or endorsed by the publisher.
