# Supplementary material for: Combined Nasal, Oropharyngeal Povidone Iodine Plus Glycyrrhizic Acid Sprays, Accelerate Clinical and Laboratory Recovery and Reduces Household Transmission of SARS-CoV-2: A Randomized Placebo-Controlled Clinical Trial
Source: Front Med (Lausanne). 2022 Apr 19;9:863917. doi: 10.3389/fmed.2022.863917 (PMC9063561; doi:10.3389/fmed.2022.863917)
Supplement: Supplementary file 1 [file Data_Sheet_1.pdf]

# Pilot study 1

## The tolerability of two different doses of povidone Iodine combined with glycyrrhizic acid as nasal and oropharyngeal sprays.

**Dr/ Hazem E Elsersy, MD, Department of ICU and anesthesia, Faculty of Medicine, Menoufia University.**

### The rationale for doing all pilots

1- The Coronavirus enters the human body via body opening mainly the nose and mouth and stays in the nasopharynx for its incubation period 5-7 days up to 14 days, where it multiplies to millions of copies. After reaching a critical number it starts to invade the lower respiratory tract in enormous numbers. Interference in this stage (early symptoms and contacts) can substantially change the outcome. Asymptomatic contacts to a known case of Coronavirus may have been infected and are in the incubation period. The early symptoms such as cough, running nose, fever, and loss of smell and taste all originate from the upper respiratory invasion from the beginning. These symptoms are alarming and represent the body's defense to limit the spread of the virus. Helping the body to get rid of the virus at this stage will result in an immediate cure.

2- Wearing masks and protective equipment aim at preventing the virus from entering the mouth or the nose. In spite of that, the virus enters because one can never wear the mask for 24 hours so, spraying antivirals in the mouth and nose will definitely reduce the viral load and help the body overcome the infection.

3- Early interference is the only hope to control the spread of the virus, the mild cases although self-limited it poses the highest risk of the virus spread as it constitutes over 80%. Furthermore, early management of mild cases prevents its transformation to severe cases.

4- The fast mutations of the virus have resulted in new strains that might show a variable degree of vaccine resistance, therefore a broad-spectrum antiviral is badly needed to overcome the vaccine-resistant virus

5- Many people including some health care professionals are under the influence of false belief that once symptoms appear that means lower respiratory invasion. This belief is unreal and upper respiratory infection can manifest in fever, cough, fatigue, running nose, loss of taste and smell, and still, lungs are preserved and can be protected if the upper respiratory infection resolves. These symptoms represent a defense mechanism by the upper respiratory system in a trial to stop the virus propagation. Helping the body at this stage to overcome the viral load would help in stopping the propagation of the virus to the lungs.

6- Glycyrrhizic acid has been shown to show high efficacy against SARS-COV2 in vitro and in vivo.

**Povidone Iodine in vitro**

1- In Vitro Efficacy of a Povidone-Iodine Nasal Antiseptic for Rapid Inactivation of SARS-CoV-2 | Global Health | JAMA Otolaryngology–Head & Neck Surgery | JAMA Network

2- Povidone-Iodine Demonstrates Rapid In Vitro Virucidal Activity Against SARS-CoV-2, The Virus Causing COVID-19 Disease | SpringerLink

3- Efficacy of Povidone-Iodine Nasal And Oral Antiseptic Preparations Against Severe Acute Respiratory Syndrome-Coronavirus 2 (SARS-CoV-2) | medRxiv

4- 0.5% povidone iodine irrigation in otorhinolaryngology surgical practice during COVID 19 pandemic (nih.gov)

**Povidone Iodine Clinical trials (published** papers and approved protocols)

1-Povidone Iodine Mouthwash, Gargle, and Nasal Spray to Reduce Nasopharyngeal Viral Load in Patients With COVID-19: A Randomized Clinical Trial | Pathology and Laboratory Medicine | JAMA Otolaryngology–Head & Neck Surgery | JAMA Network

2- PVP-I Nasal Sprays and SARS-CoV-2 Nasopharyngeal Titers (for COVID-19) - Full Text View - ClinicalTrials.gov

3- Virucidal Effect of Povidone Iodine on COVID-19 In-Vivo - Full Text View - ClinicalTrials.gov

4- COVID-19: Povidone-Iodine Intranasal Prophylaxis in Front-line Healthcare Personnel and Inpatients | ClinicalTrials.gov; 25/04/2020; TrialID: NCT04364802 | ICTRP (bvsalud.org)

5- EARLY VIRAL CLEARANCE AMONG COVID-19 PATIENTS WHEN GARGLING WITH POVIDONE-IODINE AND ESSENTIAL OILS – A CLINICAL TRIAL | medRxiv

**For information about glycyrrhizic acid safety and clinical toxicology please refer to the attached protocol**.

**Aim:** This pilot study was designed to test the tolerability of different two doses of nasal and oropharyngeal antiviral sprays utilizing two doses of both PVI and GA in twenty volunteers.

**Methods**

**Patients and design**

This study was conducted on volunteered family members and relatives including myself.

I myself, was the first human patient to receive nasal and nasopharyngeal sprays with this compound.

Twenty patients of both genders were allocated into two groups, either group1 (10 patients) received both nasal and oropharyngeal sprays with Glycyrrhizic acid (GA) 5 mg/ml concentration and povidone-iodine (PVI) in 1% concentration as active principles. Group 2 (10 human volunteers) received nasal and oropharyngeal sprays with GA 2.5 mg/ml and PVI 0.5%. The sprays have been applied 4 times per day for the full two weeks. Patients were instructed to abstain from food, drink, and smoking for 20 minutes after the application of sprays.

**Outcome measures** were blood pressure before the start of treatment, 1,3,7and 14 days after treatment, thyroid profile (TSH, T3, and T4) before the start and at the end of treatment, and tolerability of the medication. Tolerability was assessed using a 5 point-scale as follows; 1= well-tolerated, 2= tolerated, 3= mild discomfort, 4= moderate discomfort, and 5= cannot be tolerated.

**Results**

E-table1 shows the demographic data for the patients, the blood pressure measured before treatment, the blood pressure on days 1,3,7, and 14 post-treatment, and the thyroid profile. The medication was generally well tolerated. In group 1 (5 mg/ml GA+ PVI 1%) two patients out of 10 displayed throat irritation, one patient displayed throat and nasal irritation. While in group 2 (2.5 mg/ml GA + 0.5% PVI), none of the patients displayed any side effects. At the end of the treatment period, no changes have occurred to the blood pressure. with respect to thyroid profile, there was a decrease in TSH in both groups but still within normal range while no change happened with T3 or T4 (e-table1).

**Interpretation**

A compound of GA plus povidone Iodine may be safe to apply topically in the nose and oropharynx for a two-week period without notable adverse effects. It is preferred to use the low concentration of 2.5 mg/ml GA and 0.5% PVI as this concentration causes less mucosal irritation.

NB. Glycyrrhizic acid was used in the form of dipotassium glycyrrhizate for nasal and ammonium glycyrrhizate for an oropharyngeal form of a spray.

Table i. effect of PVI and GA dose on tolerability, blood pressure, and thyroid function.

| Variable | PVI 1%+ GA 5mg/ml | PVI 0.5% plus GA 2.5 mg/ml |
|---|---|---|
| Age years Mean (min-Max) | 40 (15-54) | 46 (14-68) |
| Gender Male/Female | 9/1 | 6/4 |
| Body weight KG Mean (min-Max) | 87 (65-107) | 86(67-101) |
| Pre SBP Mean (min- max) | 121.6 (109-130) | 121.8 (110-131) |
| Pre DBP Mean (min- max) | 79.5 (70-84) | 79.9 (77-84) |
| Day1 SBP Mean (min- max) | 119.5 (114-132) | 122 (116- 130) |
| Day1 DBP Mean (min- max) | 79.5 (75-88) | 81.9 (78-87) |
| Day 3 SBP Mean (min- max) | 120.3 (110-127) | 119.7 (110-132) |
| Day 3 DBP Mean (min- max) | 79.2 (70-88) | 81.4 (77-84) |
| Day 7 SBP Mean (min- max) | 120 (110-128) | 119.5 (115- 129) |
| Day 7DBP Mean (min- max) | 78.9 (70-84) | 82.1 (77-88) |
| Day 14 SBP Mean (Min-Max) | 120 (108-129) | 118 (110-127) |
| Day 14 DBP Mean (Min-Max) | 80.9 (77-88) | 81.3 (78-87) |
| Pre TSH Med. (IQR) | 2.7 (2.2-3.2) | 2.5 (2:2.9) |
| Post TSH Med. (IQR) | 1.9 (1.4-2.5) | 1.83 (1.5-2.2) |
| Pre T3 Med. (IQR) | 4.4 (4.14-4.56) | 4.04 (3.9- 4.55) |
| Post T3 Med. (IQR) | 4.09 (4.018-4.55) | 4.1 (3.86-4.58) |
| Pre T4 Med. (IQR) | 13.79 (12.24-14.95) | 13.15 (12.5-14.07) |
| Post T4 Med. (IQR) | 13.75 (12.25-14.97) | 13.82 (12.23-14.06) |
| Tolerability mean (Min-Max) | 2(1-4) | 1 (1-1) |

Table i. reveals the effect of combined PVI and GA on tolerability, blood pressure, and thyroid profile.

Pre SBP= systolic blood pressure before treatment, Pre DBP.

Normal values: TSH= 0.25 :5, T3= 2.5:8.5, T4=8:24.

## Pilot study 2

### The effect of combined povidone Iodine 0.5% with glycyrrhizic acid 2.5 mg/ml as nasal and oropharyngeal sprays on COVID-19 PCR positive patients; A pilot study.

**Dr/ Hazem E Elsersy, MD, Department of ICU and anesthesia, Faculty of Medicine, Menoufia University.**

**Rational**

1- Glycyrrhizic acid has been shown to exert a broad-spectrum antiviral activity against a wide range of viruses including Influenza A, H5N1, hepatitis A, Hepatitis C, Human immunodeficiency virus, SARS-COV virus, and SARS-COV2 virus. Povidone Iodine has been shown to rapidly inactivate SARS COV2 in vitro. Using one of these compounds alone may be insufficient to resolve the issue. PVI will rapidly inactivate the virus entering or trying to enter the cell while GA will enter the cell and prevent further multiplication of the virus. GA also inhibits Transmembrane serine protease, therefore, preventing more viruses from entering through the ACE2 receptors. The topical application to the mucus membrane mimics the invitro application on tissue culture infected with the virus.

**Aim:** Was designed to test the effect of both nasal and oropharyngeal sprays on patients with COVID-19 PCR positive patients utilizing the low dosage of combined PVI and GA in both nasal and oropharyngeal sprays.

**Recruitment**

We announced to friends and relatives that we have a new compound that possesses a potential for antiviral and that may affect the SARS-COV2 virus according to some in-vitro studies. This compound is prepared in the form of nasal and pharyngeal sprays to be applied topically and has been tested for palatability and tolerability. It may cause some irritation in an uncommon situation. Fifteen cases and five asymptomatic positive family members were recruited in the study.

**Methods**

This study is an open-label pilot study, the patients were only blinded to treatment conditions. The method of application, the expected benefit, and the expected adverse effects were extensively discussed with each patient and informed consent was taken.

The bottles were encoded by key code from 1 to 20 where odd numbers represented the treatment and even numbers represented the placebo to differentiate treatment from placebo to the examiner. Twenty pieces of paper each carrying a number from 1 to 20 were put folded in a flask. When a patient's PCR result came positive a number was selected randomly and the corresponding treatment bottles were given for his use. Twenty volunteers that were tested positive for SARS-COV2 PCR who have mild, or no symptoms were assigned into two groups, Group1 (10 patients) received the standard treatment of paracetamol, vitamin C and zinc plus the treatment sprays whereas group 2 (the control group) received the standard treatment and placebo sprays. The treatment sprays (nasal and oropharyngeal) contain a compound comprised of PVI 0.5% and GA 2.5 mg/ml, vehicle, preservative, and a flavoring for throat spray. The patients started the treatment after the confirmation of COVID positive PCR. After 4 days of treatment (4 times per day), patients were subjected to a repeat PCR on day 4 after treatment in the same center. Results of PCR were compared between the groups as number and percentage using the Chi-square test.

**Results**

Twenty patients 8 males and 12 females aged from 18-55 years, on the start of treatment all were PCR positive with variable early mild upper respiratory symptoms. Symptoms consisted of Fever (n=12), Fatigue (n=12), running nose (n=9), cough (N=10), anosmia and ageusia (N=10), or Asymptomatic PCR positive contacts (n=5). (Table2)

The placebo control group all tested positive on day 4 post-treatment while the treated group showed 50% of patients (5 patients) with negative PCR and 5 patients with positive PCR.

**Interpretation**

Once their symptoms appeared the patients came in early-stage and mild disease with absence of hypoxia, dyspnea. Patients were treated with combined antivirals (GA+PVI) to decontaminate their upper respiratory passages and reduce the viral load and virus multiplication. The PCR turned negative in 50% of patients treated on day 4. This has implications in speeding the recovery and reducing the transmission period. Further larger studies are needed to confirm this finding.

Table ii: **Age, gender, Covid symptoms, and day 4 PCR in both groups.**

| Variable | Treated | Placebo |
|---|---|---|
| Number | 10 | 10 |
| Gender M/F | 4/6 | 5/5 |
| Age (Years) Med (Min-Max) | 34(19-55) | 36(18-51) |
| Fever | 7/10 (70%) | 5/10 (50%) |
| Fatigue | 7/10(70%) | 5/10 (50%) |
| Running Nose | 5/10 (50%) | 4/10 (40%) |
| Cough | 4/10 (40%) | 4/10 (40%) |
| Anosmia Ageusia | 5/10 (50%) | 4/10 (40%) |
| Diarrhea | 1/10 (10%) | 1/10 (10%) |
| Vomiting | 1/10 (10%) | 0/10 (0%) |
| Day 4 PCR Positive | 5/10 (50%) | 10/10 (100%) |

Table ii. shows demographic data, pretreatment symptoms distribution, and PCR positive patients on day 4 post-treatment in both treated and placebo groups. M/F= Males/Females, Med (Min-Max)= Median (Minimum-Maximum). Variables are represented as numbers and percentages. Qui square test was done for PCR and revealed 50% effect of treatment on PCR on day 4 P=0.01.

## Pilot study 3

### OPD Management of Covid 19; Role of Topical Antivirals.

**Mohammed Abd-Elwahab MD 1, Zalat A PHD 2, Elgamal M, PHD 3.**

**1=Chest diseases Department, faculty of Medicine Tanta University. 2, 3 = Geology and zoology Departments, faculty of science, Tanta University**

**Purpose:** This study was conducted to detect the self-reported side effects from applying combined glycyrrhizic acid and Povidone Iodine as oropharyngeal and nasal sprays. Another objective is to test if the sprays have an effect on post-covid lost taste and smell and on the protection of household contacts of covid 19.

**Patient & Methods**

This study was designed to detect the self-reported side effects from the application of topical antivirals (Combined glycyrrhizic acid (GA) and povidone Iodine (PVI). Which was conducted on outpatients diagnosed with COVID-19 and their household contacts (N 390), long COVID 19 with unresolved anosmia, and ageusia (N 252). The ages for patients were 18:83.

This study is a nonrandomized open-label, observational pilot, 61% of the patients [n=390] were COVID positive patients and their contacts.

In total, 642 patients of both genders were subjected to the application of both nasal and nasopharyngeal sprays for two weeks. Informed consent was obtained from each patient.

**Outcome assessment**

The primary outcome of this pilot was the rate of appearance of self-reported side effects due to the use of both nasal and oropharyngeal sprays. The patients were followed up either telephonically or on regular checkup visits.

**Measurements**

Outpatient Suspected Cases of Covid-19 were Subjected to

History Taking, plain X-ray Chest, C.T Chest and Ultrasonography chest for some cases
IgG and IgM for Covid-19, PCR From Nasopharyngeal swab For Covid-19 For Some Cases
Antigen (anti-IGM) Of Covid-19 From Nasopharyngeal Swab, CRP, Ferritin, CBC, D-dimer, LDH For Some Cases, Liver& Renal Function Tests, Nasal smear for fungal study, Sputum culture and sensitivity, ECG, Echocardiography or Thallium study for some cases, Cardiac Troponin and or CKMB for some cases, CT brain for some cases, Doppler study on lower limbs for some case. Investigations were tailored according to each patient's condition.

**Group allocations and studies done**

**A-Smell and taste study (cross over study)**

- 1- GA 2.5 mg/ml +PVI 0.5%, as a nasal and pharyngeal spray as treatment at site of infection (TASI) oropharyngeal and nasal sprays were prescribed for cases and contacts (n 252).
- Oseltamivir oral capsules, vitamin C.
- **2-Group 2 (control) (n=100)**
  received corticosteroid nasal spray plus Oseltamivir oral capsules, vitamin C.

**B-Contacts study**
Family contacts were treated by a nasal and oropharyngeal spray of TASI as prophylaxis for two weeks (n=390), selected age group from 18 to 83.

**Results**

**In the smell and taste study:** 107 males and 145 females (39 % of the total sample size patients) manifested as prolonged ageusia and anosmia (long COVID category), were cross-over patients that received treatment in other centers before. cases presented by anosmia and or ageusia and treated with TASI showed gradual improvement of both taste and smell sensation within 48 hours and complete cure within one week. No side effects were reported either local or systemic. Cases in the control group control required one week to start improving and up to one month to restore smell and or taste sensation. There were no reported serious adverse effects for the use of oral and oropharyngeal sprays.

**In contacts study:** Nine patients (4% of the group) patients developed COVID symptoms with a protection rate of 96%. There were no reported adverse effects due to the use of nasal and oropharyngeal sprays.

In total, 6 patients out of 642 (1%) reported throat irritation from using the sprays while only 2 patients (0.03%) reported nasal irritation from the nasal spray. None reported allergic reactions or other serious adverse events.

**Explanation: -** It is known that Iodine is irritant to the mucous membrane, a concentration of 1% displays more frequent irritation than 0.5%. We used the 0.5% so rare irritation has been encountered. Because Glycyrrhizic acid is demulcent so, its addition has helped to reduce the irritation. Throat spray relatively resulted in more irritation, and this could be due to the addition of strong menthol as a flavoring agent

**Conclusion** the use of topical (nasal and oropharyngeal sprays) of a combination of PVI 0.5% plus GA 2.5 mg/ml in 650 patients of both genders **has no reported serious adverse effects**. This combination seems to accelerate

recovery from post-covid anosmia and ageusia and may protect household contacts from being infected with the covid-19 virus.

**recommendations**

Further randomized blinded studies are required to validate these findings.

## Pilot study 4

### New topical antiviral for protection of SARS-COV2 contacts; A pilot study

**Mohammed Mileegy Ahmed, MD, Liver institute, Menoufia University, Egypt.**

**Aim of the study.** To detect the effect of topical application (nasal and oropharyngeal) of a compound of glycyrrhizic acid 2.5 mg/ml and Povidone Iodine 0.5% (PVI) on the protection of household contacts of a PCR diagnosed early cases of COVID 19. The Compound was termed TASI (treatment at site of infection)

**Summary of methods and results**

This is a non-randomized open-label pilot study conducted at our outpatient clinic on patients coming for early symptomatic cases with a suspect of COVID 19.

PCR was done in suspected cases to confirm diagnoses. Symptoms that raise suspicion included cough, fever, running nose, Anosmia, Ageusia and may be associated with diarrhea and or vomiting and vertigo. Contacts of PCR positive patients aged 18 and above were treated with both nasal and nasopharyngeal sprays containing TASI compound 4 times per day for twenty consecutive days.

The appearance of suspected symptoms on a contact was considered as infected. The outcome of the pilot study is the percentage of contacts protected from getting COVID-19.

32 males and 18 females were contacts of Corona virus infected patients(n=19) were treated and daily followed telephonically for the appearance of COVID-19 symptoms for 21 days. Patients were given a sheet including the 21 days where each symptom can be checked in a specific day if it happened. Two contacts lost follow up. Amongst followed 48 contacts, three males and two females developed mild symptoms in the form of cough, fever and fatigue. In total 48 contacts were followed, five contacts (2 males and 3 females developed symptoms), So the total protection was about 90%.

**Interpretation**

From our experience with the rate of transmission of COVID-19 among Egyptian adult contacts an approximate of > 50% of transmission rate has been postulated. Reduction of transmission down to 10% by the use of TASI is a promising finding to control the spread of the disease. Of these 10% transmission it could be some patient did not follow the appropriate instructions or having a co-existing condition that might have resulted in this.

However, contacts getting infection (10%) developed only mild symptoms which dissipated quickly.

A major step toward the eradication of tuberculosis in the United States has been the use of isoniazid for chemoprophylaxis in certain persons who have positive tuberculin skin tests but no other evidence of active infection. Because Corona virus is a rapidly mutating virus, formation of different strains that can be vaccine resistant such as delta and omicron strains can continue for several years. Similarly, adding a chemoprophylaxis by topical antivirals may help in elimination of the pandemic.

**Opinion.** The Use of nasal and nasopharyngeal GA 2.5 mg/ml plus PVI 0.5% is a promising treatment that appear to control the spread of corona virus among the household contacts and generalization of its use can help with the vaccine in ending up this pandemic and eradicate this virus.

**Procedure of Glycyrrhizic acid, ammonium glycyrrhizate, potassium glycyrrhizate and other salts extraction from licorice; A new economic technique.**

**Magy A.H. Zahran.**

**Professor of organic chemistry, faculty of science, Menoufia University.**

The following represents the main steps of this novel non-toxic and economic method of glycyrrhizic acid and ammonium glycyrrhizate extraction. The details of know how supplemented by videos, the details of the preparation of novel salts, and details of HPLC analysis will be available after publication.

**Heating step:**

*Weigh 1 kg of a well-crushed licorice.

*Add 10 L of water.

* Heat at 40 °C to below 60 °C for 3.5 hrs.

*Filter the hot extract soln.

**Precipitation step:**

*Acidify the solution with HCl to obtain PH 3.5.

the ratio is 30 ml 33% HCl for each 1 L of extract or till obtain PH of 3.5.

*Stir the extract during acidification continuously for 30 min.

*Leave it for 2 hr. till all Glycyrrhizic acid settle down.

*Decant the supernatant then filter the ppt off, wash with cold water.

* Dry the ppt well then weight it (22) grams, then detect the melting point at (214-218) °C.

**Conversion of Glycyrrhizic acid to Monoammonium Glycyrrhizinate:**

*Dissolve (22) grams of Glycyrrhizic acid in (330) ml of hot water at (50 :60) °C.

*During heating, add (20) grams of Ammonium carbonate with stirring (bubbles will appear).

*After accomplishment of the ebullition, evaporate the resultant soln. to get crystals of Monoammonium Glycyrrhizinate.

* Dry it well then weigh it (20) gm, Detect melting point at (218-220) °C.
